# Isolation of Human Small Extracellular Vesicles and Tracking of Their Uptake by Retinal Pigment Epithelial Cells In Vitro

**DOI:** 10.3390/ijms21113799

**Published:** 2020-05-27

**Authors:** Irene C. Marcu, Naja Eberhard, Anaïs Yerly, Verena Balmer, Andrew Hemphill, Helga Mogel, Véronique Gaschen, Michael H. Stoffel, Jasmin Bluteau

**Affiliations:** 1 Division of Veterinary Anatomy, Vetsuisse Faculty, University of Bern, 3012 Bern, Switzerland; irene.marcu@gmail.com (I.C.M.); anais.yerly@dbmr.unibe.ch (A.Y.); helga.mogel@vetsuisse.unibe.ch (H.M.); veronique.gaschen@vetsuisse.unibe.ch (V.G.); michael.stoffel@vetsuisse.unibe.ch (M.H.S.); 2 Institute of Parasitology, Vetsuisse Faculty, University of Bern, 3012 Bern, Switzerland; naja@klaf.ch (N.E.); verena.balmer@vetsuisse.unibe.ch (V.B.); andrew.hemphill@vetsuisse.unibe.ch (A.H.)

**Keywords:** small extracellular vesicles, BODIPY, PKH, EV, retinal pigment epithelium, *T. gondii*

## Abstract

Small extracellular vesicles (EVs) are among the most frequently investigated EVs and play major roles in intercellular communication by delivering various cargo molecules to target cells. They could potentially represent an alternative delivery strategy to treat ocular toxoplasmosis, a parasitosis affecting the retinal pigment epithelium (RPE). To date, the uptake of human small EVs by RPE cells has never been reported. In this study, we report on the intracellular uptake of fluorescently labelled human urine and fibroblast-derived small EVs by human RPE cells. In summary, both dye-labelled urinary small EVs and small EVs obtained from fibroblasts stably expressing membrane-bound green fluorescent protein were successfully internalized by RPE cells as revealed by immunohistochemistry. In recipient ARPE19 cells, BODIPY-labelled small EVs were found in close vicinity to the parasite *Toxoplasma gondii.* Additionally, an ultrastructural method was enabled to distinguish between labelled exogenous and endogenous small EVs within target cells.

## 1. Introduction

Ocular toxoplasmosis (OT) is the most frequent cause of retinochoroiditis worldwide, and it can lead to partial or complete loss of vision. Unfortunately, parasites are able to persist within host cells and repeated infections may occur, thus making treatment difficult. Novel approaches such as the use of nanomedicine to specifically deliver antiparasitic drugs might pave the way to a new anti-parasitic therapy [1]. To date, liposomes, solid lipid nanoparticles, and polymer nanoparticles are under investigation as potential vehicles for antiparasitic drug delivery. The first results are encouraging, and they have prompted us to investigate small extracellular vesicles (EVs) as yet another previously neglected drug delivery taxi. The use of small EVs aims at improving absorption and targeting and at reducing systemic drug concentrations by means of a drug carrier that is safe, inexpensive and reliable.

Small EVs are extracellular vesicles in the range of 40 to 150 nm that are secreted by cells and are abundant in all biofluids, including blood and urine [2]. Small EVs can be isolated using differential centrifugation and precipitation, resulting in co-purification of non-exosomal proteins and size-based separation. Additionally, the current enthusiasm in exosome research has led to the publication of a variety of different protocols for vesicle isolation and characterization, making reproducibility cumbersome.

Regardless of their origin, all exosome membranes express annexin and tetraspanin transmembrane proteins such as CD63, CD9, and CD81. Tetraspanins regulate cell signalling, cellular mobility, and protein trafficking. They provide a means to identify isolated small EVs by immunolabelling or by specific tagging using fluorescent reporter proteins.

Previously described as cellular garbage [3,4], small EVs are now considered to play important physiological roles in intercellular communication by transferring lipids, proteins, and nucleic acids to acceptor cells. By delivering their cargo, small EVs influence cellular protein expression in recipient cells. Transferred mRNA can undergo translation, and miRNA can regulate many cellular processes such as the cell cycle, cell differentiation and apoptosis.

The ability to transport and deliver cargo to intracellular compartments of the acceptor cells indicates that small EVs may be potentially exploited for therapeutic purposes such as drug delivery or targeted gene therapy [5]. Small EVs are endowed with uniquely favourable properties, such as superior biocompatibility, absence of immunogenicity, small size, low toxicity, natural targeting capability, and the ability to cross the blood-brain barrier. These are properties that other nanovesicles (such as nanoparticles) are mostly lacking. All these properties can be leveraged to use small EVs as biomarkers, therapeutic drug carriers, or delivery vehicles [6]. Targeting small EVs to specific cells would further improve their use as therapeutic vehicles.

In the mouse, for instance, small EVs from dendritic cells expressing a fusion protein of lysosomal-associated membrane protein-2 (LAMP2) and a central nervous system-specific rabies glycoprotein virus peptide were used to deliver siRNA to the brain via systemic injection [7]. 

To use small EVs as potential drug delivery vehicles [8], it is important to develop and further improve drug-loading protocols. To this end, small EVs may be treated by simple incubation with membrane-permeable agents (e.g., curcumin, dopamine, or doxorubicin) or by electroporation to load hydrophilic membrane-impermeable drugs (and miRNA and siRNA) [9]. It is important to determine the exact intracellular localization of small EVs that have reached their target cells. Furthermore, methods specifically inducing the release of these drugs must be developed [9,10].

Many studies have relied on labelled small EVs to track these nanocarriers and their fate both in vivo and in vitro. Some of the most frequently used lipophilic dyes belong to the PKH family [11], named after their discoverer Paul Karl Horan. Unfortunately, PKH labelling can result in a vesicle size shift [12], which might impair both cellular uptake and in vivo biodistribution, with larger particles preferentially being trapped in the liver and spleen [13]. As for PKH dye labelling, false positives were reported in uptake studies when using the dye-only controls. [11,12]. Concerning the choice of the dye boron-dipyrromethene (BODIPY), many studies use BODIPY TR ceramide that has the same stereochemical conformation as natural biologically active sphingolipids. Like PKH dyes, BODIPY FL belongs to the family of hydrophobic dyes that stain lipid membranes. 

A second strategy for the fluorescent labelling of small EVs is the use of gene-editing technology. A fluorophore such as green fluorescent protein (GFP) or luciferase is then fused to a marker protein (such as CD63) on the exosome membrane, thus enabling the tracking of small EVs in live imaging [14]. This imaging strategy avoids potential false positive signals originating from unbound dye or other labelled lipids. Unfortunately, this approach may only be used for small EVs that are isolated from cells in suspension or culture and not small EVs collected from biological fluids such as human urine samples. 

Alternatively, the use of synthetized gold nanoparticles as contrast agents for computed tomography (CT) is a suitable method for labelling and in vivo tracking of administered small EVs. 

In this study, we investigated whether human primary and immortalized retinal pigment epithelium (RPE) cells internalize small EVs. Human urinary small EVs and small EVs released from human fibroblasts were isolated using ultrafiltration. All the small EVs were analysed with respect to size distribution and ultrastructure (TEM). In addition, the protein profile of the urinary small EVs was assessed. Beyond, we pursued the objective to visualize the uptake of fluorescently labelled small EVs at the ultrastructural level by means of photooxidation, thus extending current knowledge beyond light microscopy. This information is essential for gaining integrative insight into the downstream fate of internalized small EVs and will help to identify whether drugs are successfully released intracellularly. RPE cells were selected as target cells to study exosome uptake because they are a relevant therapeutic target for the treatment of ocular toxoplasmosis. 

## 2. Results

### 2.1. Isolation, Characterization and Labelling of Human Urinary and Fibroblast-Derived Small EVs

**Figure 1:**
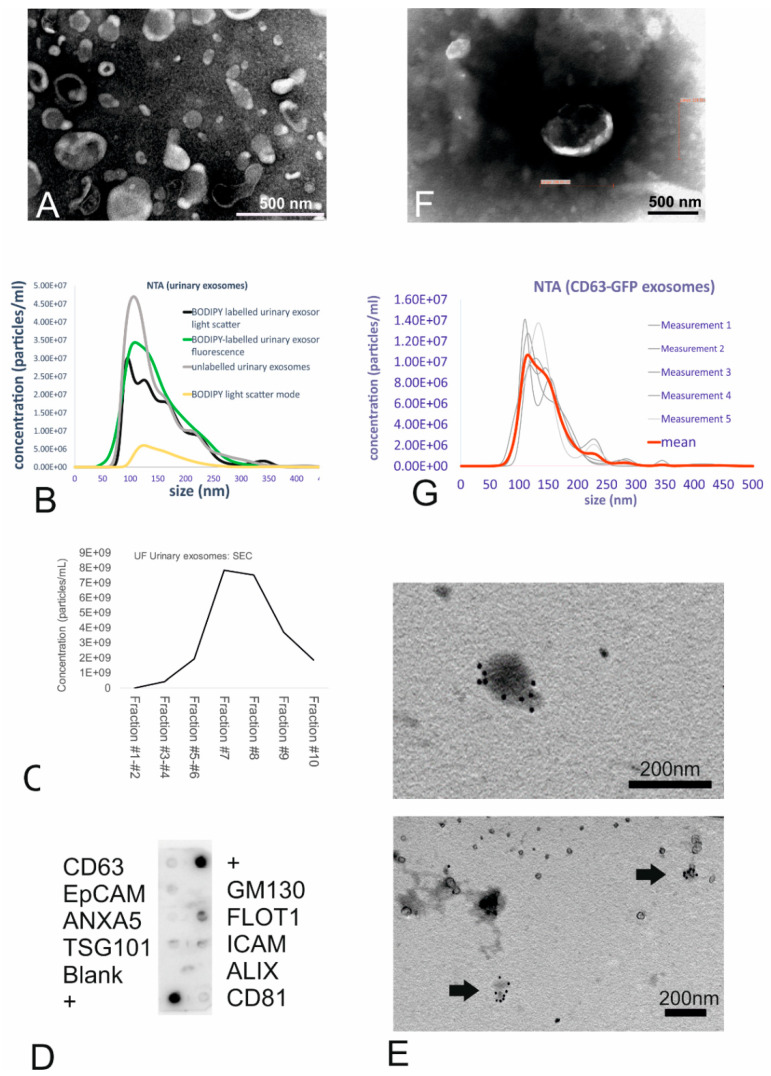
Isolation, characterization, and labelling of the urinary small extracellular vesicles (EVs). (**A**) TEM images of the small EVs obtained using ultrafiltration (UF). UF samples showed intact cup-shaped double-membrane structures. Scale bar = 0.5 µm. (**B**) Nanoparticle tracking analysis (NTA) showing the size distribution of the urinary small EVs (both unlabelled and BODIPY-labelled). The graph shows the concentration on the Y-axis and size distribution on the X-axis. (**C**) Size-exclusion chromatography of unlabelled urinary small EVs revealed small EVs (peaks in the particle count graph) in fractions 6–8. (**D**) Exo-Check antibody array showing the presence of the following exosomal markers in the ultrafiltrated small EV urinary samples: FLOT1, ICAM, TSG101, CD81, and CD63. (**E**) Immunogold TEM (urinary small EVs) showing the presence of CD63 tetraspanin, an exosomal marker, in vesicles in the UF sample. (**F**) TEM of the ultrafiltrated medium collected from the CD63-GFP transduced human fibroblasts shows some structures displaying typical exosome features. Scale bar = 0.5 µm. (**G**) NTA of the genetically tagged fibroblast-derived small EVs: Analysis displays the light-scatter results (small EV mean size of 146 nm).

Urinary small EVs were isolated from human morning urine using an ultrafiltration (UF) method consisting of a combination of low-speed centrifugation, filtration, and concentration. Transmission electron microscopy (TEM) corroborated the spherical cup shape, double membranes, and size range consistent with membrane-bound vesicles, which were identified as small EVs based on size (Figure 1A). Nanoparticle tracking analysis revealed unlabelled urinary small EVs to constitute a suspension of small-sized vesicles with a size range of 102 to 198 nm (mean size: 146 nm, see Figure 1B, grey line).

After labelling of the urinary small EVs with the fluorescent dye BODIPY FL C16, mean vesicle size was 155 nm as determined in light scatter mode (Figure 1B, black line), indicating no size shift due to the labelling procedure. In the light scatter mode, nanoparticle tracking analysis (NTA) revealed particles in the dye-only control (Figure 1B, yellow line) as well indicating that the dye alone exhibited a light-scattering behaviour, resulting in a highly uniform size distribution. The efficiency of labelling the small EVs with BODIPY was 100% as revealed by NTA using the fluorescent mode (Figure 1B, green line) when comparing the concentration and size distribution to the black line (light scatter of labelled small EVs). 

For the purpose of intracellular exosome tracking, purity did not play a major role. However, purification processes such as size-exclusion chromatography (SEC) confirmed the presence of small EVs due to the size-related elution in certain fractions. Indeed, the highest yield of small EVs (with a mean size of 192 nm as revealed by NTA) was found in fraction 7, with vesicles eluting predominantly in fractions 7, 8, and 9 (Figure 1C), as predicted for small EVs.

At the protein level, characterization with an antibody array revealed that the urinary small EVs were positive for Flot1, ICAM, Tsg101, and CD63 proteins and moderately positive for CD81 (Figure 1D). The absence of reactivity with an antibody against GM130 (indicating the absence of contamination with cellular debris) also demonstrated the purity of the exosome fractions. Immunoelectron microscopy with the anti-CD63 antibody corroborated the membrane-based presence of tetraspanin on the outer surface of microvesicles, which exhibited the typical morphology of small EVs (Figure 1E), in urinary small EV samples. 

As a second strategy to generate fluorescent small EVs, human primary fibroblasts stably expressing GFP-tagged CD63 were generated by transduction with a lentivirus carrying the copGFP-fusion protein. Transfected HEK293T cells successfully expressing CD63-GFP were used for lentivirus production. Following virus titration, transduction of fibroblasts (Supplementary Figure S1) and three subsequent passaging events, the medium isolated from the cultured CD63-GFP-transduced human fibroblasts was collected and ultrafiltrated. The concentrate was analysed for ultrastructure (Figure 1F, TEM) as well as for microvesicle size (Figure 1G). TEM revealed that GFP positive fibroblast-derived small EVs were typical cup-shaped membranous particles (Figure 1F). Although obtained concentration was very low, the NTA particle tracking confirmed the presence of small-sized vesicles with a mean size of 146 nm (Figure 1G). Refractive index 3D plots of size versus intensity versus vesicle concentration showed the small EVs to be monodisperse fraction, although there were significant differences in size, intensity, and concentration of the vesicles (Supplementary Figure S2A). On the protein level, the expression of Tsg101 and ICAM was detectable (Supplementary Figure S2B); NTA, using the fluorescence mode, confirmed the presence of GFP fluorescent vesicles (Supplementary Figure S2C).

Taken together, the ultrastructural data of small EVs derived using ultrafiltration from urine and from the supernatant of transduced human fibroblasts provided convincing evidence that the isolated vesicles were small EVs and that the small EV labelling strategy was successful.

### 2.2. In Vitro Uptake of Labelled Small EVs

**Figure 2:**
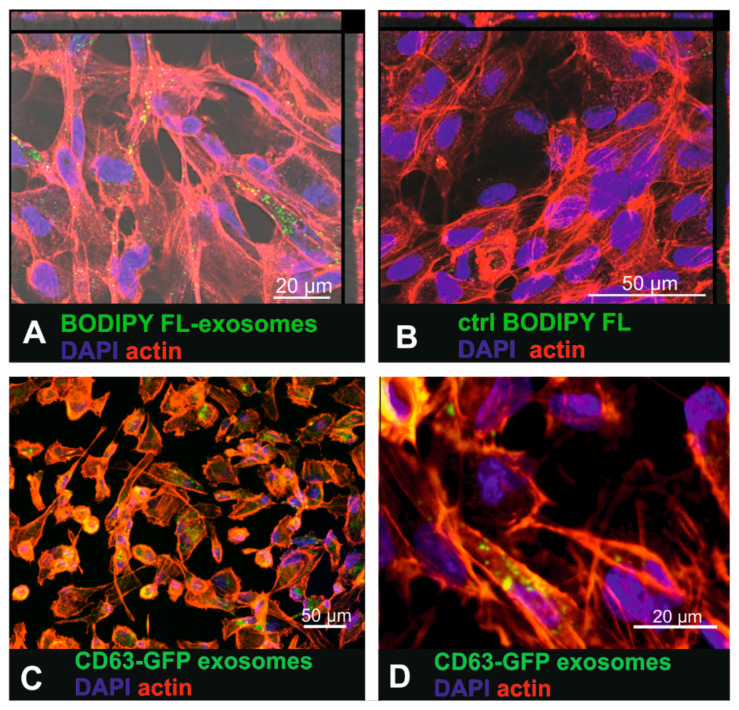
Uptake of urinary BODIPY-labelled small EVs and CD63-GFP tagged fibroblast-derived small EVs in ARPE19 cells. (**A**) Incubation of the ARPE-19 cells with BODIPY-labelled small EVs that were purified to remove unbound dye. (**B**) ARPE-19 cells exposed to purified (removal of small-sized vesicles) BODIPY solution (control (ctrl)) where no internalized fluorescence should be detected. (**C**) Uptake of fibroblast-derived CD63-GFP tagged small EVs in ARPE19 cells. (**D**) Higher magnification of Figure 2C showing intracellular CD63-GFP expression that visualized tagged internalized small EVs.

To investigate whether human RPE cells are able to internalize labelled small EVs, the primary RPE cell line ARPE19 was exposed to BODIPY FL C_16_-labelled urinary small EVs as well as to CD63-GFP tagged fibroblast-derived small EVs (Figure 2). Sixteen hours after exposure of ARPE-19 cells to the BODIPY FL C_16_-labelled urinary small EVs, intracellular fluorescent punctate signals were observed (Figure 2A). Interestingly, the exposure of the cells to the purified dye-only control solution resulted in a low number of intracellular dots (Figure 2B) as well, indicating that dye was not completely removed in the purification column. Yet, these findings indicated that the most of the intracellular signal represented BODIPY-labelled small EVs and not unbound dye. 16 h after incubating ARPE19 cells with BODIPY-labelled urinary small EVs, lysotracker co-localization revealed occasional co-localization of exosomal and lysosomal tags (arrows in Supplementary Figure S3A,B), indicating some extend of sorting to the lysosomes. Using Nomarski differential interference contrast microscopy, the fluorescent labelled small EVs were visualized within larger contrast rich intracellular vesicles (endocytotic vesicles, arrows in Supplementary Figure S3C,D).

Small EVs isolated from the CD63-GFP lentivirus-transduced human fibroblasts also accumulated within the cytosol of the ARPE-19 cells (Figure 2C,D). However, the fluorescence was much dimmer than it was in the cells that had internalized the dye-labelled small EVs.

**Figure 3:**
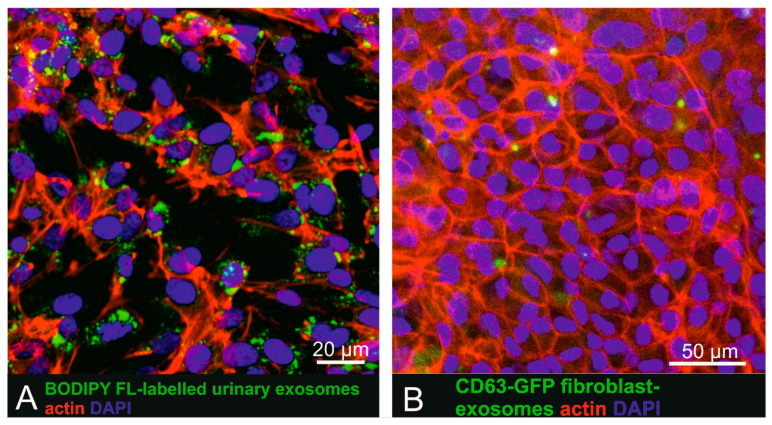
Uptake of urinary BODIPY-labelled small EVs and CD63-GFP tagged fibroblast-derived small EVs in human iPSC derived RPE cells. (**A**) Incubation of iPSC-derived RPE cells with BODIPY-labelled small EVs. (**B**) iPSC-derived RPE cells exposed to CD63-GFP fluorescent tagged primary fibroblast-derived small EVs also showed intracellular exosome localization.

**Figure 4:**
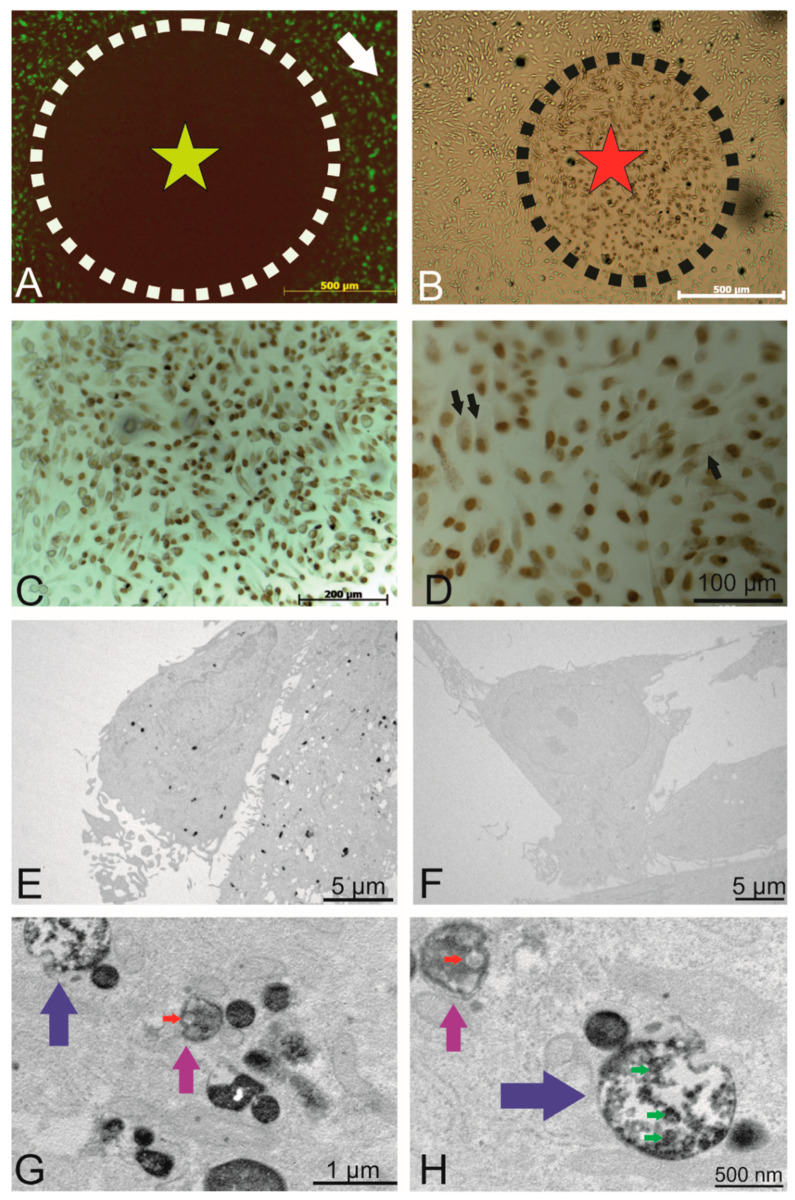
Intracellular localization of the internalized PKH-labelled small EVs. (**A**) Circled area shows the area of fluorophore (label of the small EVs) following photobleaching (outside this circle, there was no light exposure). (**B**) DAB precipitate was observed in areas of photobleaching. (**C**) Photobleached area where a precipitate was observed. (**D**) Depiction of the photobleached areas where the nucleus was visible under different magnifications (Draq5 that was photoconverted), and the fine intracellular precipitate dots represent the PKH-labelled small EVs. (**E**) TEM micrographs showing the DAB precipitate representing labelled small EVs as electron dense dots. (**F**) Representative non-photobleached areas where no precipitate could be observed (negative control). (**G**) Higher magnification of the TEM micrographs. Labelled small EVs are visible within endosomes (magenta arrows) and multivesicular endosomes (blue arrows). (**H**) A second representative micrograph showing multivesicular endosomes (blue, magenta arrows) containing small EVs. The red arrow indicates DAB-negative small EVs (size of about 100 nm). Green arrows point to DAB-positive vesicles of similar size.

To investigate uptake of labelled small EVs by pigmented cells more closely mimicking physiological RPE cells, BODIPY FL C_16_-labelled small EVs and CD63-GFP tagged human fibroblast-derived vesicles were also administered to pigmented human iPSC-derived RPE cells (Figure 3). Recipient cells displayed a cobblestone-like pattern characteristic for RPE cells and also expressed occludin as a tight-junction marker (Supplementary Figure S4). iPSC-derived RPE cells, following incubation for 16 h with urinary (Figure 4A) or fibroblast-derived (Figure 4B) fluorescent human small EVs showed intracellular fluorescent dots, indicating successful cellular uptake.

### 2.3. Intracellular Localization of the Internalized Small EVs

To investigate the exact intracellular fate of the internalized small EVs (organelles, intracellular localization), we made use of the photooxidation technique. This method allows us to reveal the presence of a fluorophore (in this particular case, the lipophilic dye PKH67 of dye-labelled small EVs) in both light and electron microscopy. This technique makes us of the fluorescent signal to oxidize DAB. Prior to photooxidation, NTA was performed on dye-only controls, unlabelled and PKH-labelled urinary small EVs in both the fluorescence and light-scatter mode. Unlabelled urinary small EVs were non-fluorescent (see Supplementary Figure S5). There was no size shift detectable with PKH labelling (red line compared with grey line). Fluorescence of labelled was detectable (light green line in Supplementary Figure S5), although the measured concentration under fluorescence mode was lower than under light-scatter mode (red line). As a control, PKH dye particles alone scattered light showing a mean particle size of 90 nm (lower size than the median size of urinary small EVs) and were dimly fluorescent (dark green line, Supplementary Figure S5).

To this end, areas of interest were exposed to an intense light source (X-cite XYLIS), resulting in the generation of DAB precipitates at the expense of fluorescence (Figure 4A, black circle). The presence of DAB precipitates was detected under the light microscope after no more than 10 min of illumination (brown cells within the dashed line in Figure 4B marked with a red star). Figure 4C provides an overview of the illuminated region with intense nuclear DAB precipitate (Draq5 was used as a fluorescent nuclear counterstain). At a higher magnification (Figure 4D, black arrows), a dotted DAB precipitate is visible within most cells.

TEM revealed the presence of DAB precipitates in the cytoplasm (Figure 4E), whereas in non-photoconverted control areas of the cell culture, no DAB precipitate was observed (Figure 4F), indicating that the photoconversion of DAB did not result from exposure to ambient light. Figure 4G,H depicts views at higher magnification, in which DAB precipitates were observed within endosomes (Figure 4G,H, magenta arrows) and multivesicular endosomes (MVEs, Figure 4G,H, blue arrows). Red arrows point to DAB-negative endogenous small EVs with a size of approximately 100 nm, whereas green arrows indicate DAB-positive labelled vesicles with a similar size. In summary, photooxidation unveiled PKH-labelled endosomes, confirming the presence of internalized, labelled small EVs in endosomal structures.

### 2.4. Uptake and Localization of Urinary Small EVs in Toxoplasma gondii (T. gondii)-Infected Cells

**Figure 5:**
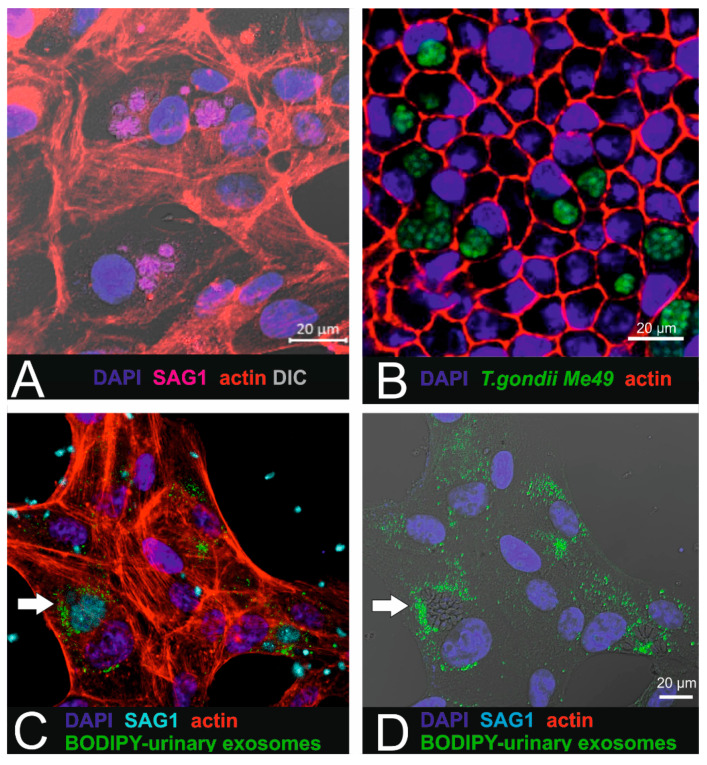
Combination of parasite infection with urinary exosome uptake in RPE1 cells. (**A**) *T. gondii*-infected ARPE-19 cells. SAG-1 immunoreactivity reveals the presence of the parasites. (**B**) In pigmented iPSC-derived RPE cells (cobblestone morphology), intracellular localized parasites (*Me49 T. gondii*) are arranged in cysts. (**C**,**D**) Micrographs showing the internalized BODIPY-labelled urinary small EVs (green) localized in close proximity to the parasites (cyan: SAG1 parasite surface antigen). Target cells were ARPE19 cells.

In the next step, intracellular localization of internalized labelled small EVs was investigated in ARPE-19 cells infected with *T. gondii* tachyzoites (Figure 5).

For this purpose, ARPE-19 cells were first infected with parasites (Figure 5A,B) as described by others [15], resulting in the presence of tachyzoite-containing parasitophorous vacuoles (PVs) within the cytoplasm. After establishing the in vitro infection model, BODIPY-labelled urinary small EVs were added to parasite-infected ARPE19 in order to investigate intracellular uptake in affected recipient cells. Intracellular localization was observed using confocal imaging (Figure 5C,D). Interestingly, the labelled small EVs were found in close proximity to the intracellular parasites and accumulated in the peripheral region of the parasitophorous vacuoles.

## 3. Discussion

The goal of this study was to assess whether human urinary and human primary cell-derived small EVs are internalized by human RPE cells. In addition, in view of the potential application of small EVs as drug delivery vehicles to target retinal or CNS tissue, we studied the uptake and distribution of urinary small EVs in the retinal cells infected with the apicomplexan parasite *T. gondii*, the causative agent of ocular toxoplasmosis affecting both the brain and retina.

Small EVs were collected from urine using ultrafiltration before being fluorescently labelled. As an alternative, small EVs isolated from the supernatant of human fibroblasts were genetically labelled with CD63-GFP. Both sources are suitable in terms of a potential clinical application in the future. Both isolation techniques yielded particles in the expected size range of small vesicles (approximately 50–200 nm). The presence of specific marker proteins (cells positive for CD81, CD63, and Alix) along with the absence of GM130 as a cytosolic marker confirmed the purity of the isolated urine preparations. Size-exclusion chromatography (SEC) confirmed that exosomal fractions are eluted predominantly in fractions 7 and 9. Concerning the potential labelling strategies, many approaches are currently followed, depending on the application. When added to cells in culture, BODIPY dye enters the lipid pathway, is transferred into phospholipids, and is finally integrated into the lipid bilayer [16]. BODIPY micelle formation has not been observed as reported for the PKH dye [11], but the dye incorporates into all membranes, including those of internalized small EVs. While our experiments showed that BODIPY is an ideal dye to label lipids, membranes, and other lipophilic compounds and that it provides a strong fluorescence signal, it was also evident that the uptake of the purified dye control resulted in intracellular staining dots, complicating the analysis of the exosome uptake studies. The use of column purification in combination with swinging bucket centrifugation reduced the proportion of intracellular fluorescent dots in the target cells to 10%. Thus, some false positive signals were unavoidable. Furthermore, rapid photobleaching was observed, thus precluding the use of BODIPY for photooxidation procedures where we used PKH67. According to [17], this problem might be circumvented by the use of the BODIPY ceramide dye [18], an approach we have not pursued further.

Although BODIPY-labelled small EVs could be tracked using NTA, their fluorescence was dim, thereby limiting quantitative analysis. This limitation might be overcome with other tools, such as ImageStream and fluorescence-conjugated antibodies to stain for exosomal surface markers [19] or by relying on NanoFACS of carboxyfluorescein diacetate succinimidyl ester-labelled small EVs [20].

We chose fibroblasts as an exosome source in view of future applications because fibroblasts are proliferative with a low level of senescence induction (only at later passages). This is why they are ideally suited as primary cells to shed genetically labelled small EVs carrying a CD63-GFP tag. CD63-GFP-positive small EVs also circumvent potential problems associated with post hoc labelling. This cell line produced CD63 tetraspanin-GFP-tagged small EVs with a mean size of 146 nm that were successfully taken up by the target ARPE-19 cells. This opens the door to assess loading the small EVs with anti-parasitic drugs as a next step. For future clinical application, the use of animal-origin factors in the cell culture and the minimization of risks associated with lentivirus transduction should be addressed as potential safety issues.

Although the precise uptake mechanisms and intracellular fate of small EVs remain to be elucidated, the present findings support the contention that, in principle, drugs may be delivered to RPE cells by using either urinary or fibroblast-derived patient-specific vesicles.

These findings open up new perspectives for the treatment of eye infections such as OT but potentially also pave the way towards the use of small EVs to treat dry age-related macular degeneration. To date, systemic therapy in OT is poorly tolerated and allows only transient parasite eradication but does not eliminate the slowly proliferating parasite bradyzoites encapsulated in tissue cysts. As drugs acting on cysts have recently become available [21], targeted therapy would obviously be an attractive option. However, tracking small EVs in vivo remains a challenge. Dye-based imaging relying on near-infrared dyes or bioluminescence, which is currently being investigated, and each of the possibilities has pros and cons [22,23]. A promising alternative consists of nuclear imaging (3D positron emission tomography) after radionuclide labelling with ^99m^Tc-hexamethylpropyleneamineoxime (HMPAO) [23]. These authors were able to capture the desired clearance rate and achieve the accumulation of intravenously injected small EVs in tumours. Alternatively, small EVs may also be loaded with an MRI contrast agent such as ultrasmall super paramagnetic iron oxide (USPIO). For example, vesicles loaded with USPIO nanoparticles were shown to travel from the injection site (foot pads) to regional popliteal lymph nodes [24].

Drug vehicles such as nanoparticles (gold, silver), liposomes, and small EVs are small enough to cross cell barriers and can modulate target cells. In toxoplasmosis, silver, platinum, and gold nanoparticles were shown to be attractive drug carriers because of their antimicrobial and antiparasitic effects. They were described to accumulate in specific tissues and to act on the replication stages of *T. gondii* through interactions with reactive oxygen species (ROS) [25]. The tendency of nanoparticles to accumulate in specific tissues supports the idea of using nanoparticles to target parasite cysts in the host tissue. Such particles were synthesized in combination with extracts of natural plants (date palm and nabka leaves) and were tested and compared to alternative sulfadiazine (oral: 100 mg/kg) drug therapy [26], with the outcome showing that the treated nanoparticles prevented the occurrence of hepatotoxicity more effectively than did standard treatments.

In another study, two different drugs against ocular toxoplasmosis were successfully loaded into polystyrene latex particles during polymerization, generating core–shell nanoparticles [27]. However, the drug-loaded particles were no more effective than the unloaded particles.

Orally administered triclosan-loaded liposomes have also been investigated in terms of their efficacy against a virulent strain of *T. gondii* [28]: Both the drug alone and drug-loaded liposomes reduced parasite burden and infectivity of tachyzoites harvested from the peritoneal fluid of infected treatment groups, and the tachyzoites caused disintegration of plasma and nuclear membranes and vacuolization in the cytoplasm.

Altogether, ideal carriers should be non-toxic, non-immunogenic, well-tolerated, and effective. To date, these criteria are best met by small EVs, since nanoparticles still raise concerns about cytotoxicity, low drug-loading capacity, and insufficient delivery. Small EVs can also be successfully loaded with drugs such as anticancer therapeutics as described in [29], where small EVs obtained from a macrophage cell line are loaded with paclitaxel and doxorubicin by mixing and subsequent electroporation or sonication. To date, the only in vivo study on small EVs for use against toxoplasmosis involved the development of a vaccine against congenital toxoplasmosis in mice, with the small EVs obtained from a splenic dendritic cell line pulsed with *Toxoplasma* antigens [30].

These data are expected to provide a reliable basis for further studies dealing with drug loading and with assessing the anti-parasitic action on infected ARPE-19 cells. Interestingly, our results showed that internalized small EVs are localized in close vicinity to the parasites in the ARPE-19 cells, thus making them a promising tool for targeted drug delivery. One of the major concerns in drug delivery is whether the drugs can be released sufficiently, i.e., in therapeutic amounts. The regular fate of the small EVs after their uptake by energy-dependent endocytic internalization is fusion with lysosomes. To promote drug delivery, premature lysosomal degradation of small EVs must be prevented. This prevention may be achieved by modifying the small EVs with unsaturated dioleoyl phosphatidylethanolamine (DOPE), which triggers the fusion of the liposomal membrane with the endosomal membrane, releasing the drug into the cytoplasm. In [31], this combination of a nanoparticle with pH-sensitive DOPE was described and indicated that the DNA was released into the cytoplasm during gene transfection.

Prior to therapeutic applications, more work will obviously be needed to shed more light on the current black box; that is, researchers must investigate uptake mechanisms, the intracellular fate and the release of cargo from drug-loaded small EVs. In one study, the intracellular fate of labelled small EVs was tracked by live imaging using lysosomal markers (LysoTracker) and ER trackers as described in [32], and it was shown that the small EVs entered the cells via filopodia, were sorted in endocytic vesicles and targeted to the ER before reaching the lysosome. Within 16 h post-labelling, we did not observe substantial colocalization with lysotracker or LAMP-1 (not shown). A time course (high resolution microscopy) using lysosomal markers and ER trackers will shed more light on the time-course of exosome uptake events such as early endosomal localization (EEA-1), late endosomes (CD71 as a marker), and lysosomal degradation (LAMP1). 

The lack of ultrastructural localization of endocytosed fluorescently labelled vesicles in the current literature is probably due to the challenge of differentiating between endogenous nonfluorescent and internalized fluorescent microvesicles using TEM. As demonstrated in the present study, photooxidation is a valuable tool to visualize labelled small EVs at the ultrastructural level. Transmission electron microscopy revealed labelled and non-labelled endogenous small EVs inside vesicles with a double membrane. The fine precipitate resulting from unbound PKH should be distinguished from the specific DAB pattern [33]. Photooxidation thus allows the differentiation of endogenous small EVs from exogenous, labelled small EVs. A considerable number of preliminary experiments, however, showed that this technique only yields positive DAB precipitates when adequate fluorochromes and light sources are used. The protocol needs to be adapted to both the fluorochrome and the objective used since the intensity of the DAB precipitation is related to the objective and to the singlet oxygen generated by the fluorophores, rather than to brightness alone. Hence, a non-optimal setup will fail to produce any DAB precipitate, a failing that we experienced in our first attempts.

This need for an optimal setup was also reflected by the fact that there is only one other study in which photoconversion was performed to visualize endocytosed small EVs [34]. In this study, the authors described the internalization of exogenous small EVs isolated from mouse brains by a second neuron that was then passed on to a third neuron. For the photoconversion, they used FM1-43FX fixable membrane stain as a label, and the dye reacted with DAB to form an insoluble precipitate, which revealed endosomes as dots smaller than 100 nm.

Most photoconversion studies describe the importance of the temperature during the cooling stage and the additional oxidation of the DAB solution by bubbling oxygen into the solution [35,36] as key factors for successful photoconversion. Herein, we conclude that, at least for the PKH fluorophore, a strong light source (such as the X-City XYLIS illumination system), at best in combination with the right objectives and adequate cooling of the sample (as a function of the exposure time), are the key elements to obtain a positive DAB precipitate.

In summary, this study shows for the first time that ARPE19 and iPSC-derived RPE cells are internalizing human small EVs, both on the light-microscopic as well on the ultrastructural level. 

These results should pave the way for further investigations addressing the targeting, fate of drug-loaded small EVs and the release of their content. Further steps involve the combination of parasite infection and the administration of drug-loaded small EVs and the investigation of whether there is a reduction in parasite burden in comparison to treatment alone. Successful loading of small EVs with drugs requires further confirmation using chemical methods. The question of whether any loading strategy (such as electroporation) disrupts the integrity, stability, function, and loading efficiency of the vesicles also awaits to be clarified. 

Further steps will also address the questions regarding large scale production of small EVs and their clinical use to treat brain disorders. Taken together, this study encourages the development of small EVs as a parasitic drug delivery vehicle with the core concept of designing an OT treatment strategy to lower systemic drug concentration while obtaining optimal local concentrations. Small EVs represent great candidates for targeted drug delivery by ensuring absorption, long-circulation time, and targeting along with a low or even missing toxicity. 

## 4. Materials and Methods

### 4.1. Cell Culture

The ARPE-19 cell line (ATCC CRL2302), a human retinal pigment epithelial cell line, was cultured in maintenance medium (ARPE-19 medium) containing 10% foetal bovine serum (FBS, Gibco LOT 42Q7362K) and 1% penicillin/streptomycin (P/S) in high-glucose DMEM, and GlutaMAX, supplemented with pyruvate (Thermo Fisher Scientific, Waltham, MA, USA). When 80% confluent, the cells were split 1:4 using the TrypLE Express cell dissociation enzyme (Thermo Fisher Scientific). Additionally, human-induced pluripotent stem cells (SC102A-1-GVO-SBI, Systems Biosciences, Palo Alto, CA, USA) were cultured in E8 maintenance medium (Thermo Fisher) on vitronectin-coated dishes and dissociated for differentiation as RPE cells according to [37]. Sonic Hedgehog was obtained from Bio-Techne (Abingdon, UK), basic fibroblast growth factor was obtained from PeproTech (London, UK), and retinoic acid was obtained from Sigma Aldrich (Buchs, Switzerland). Following differentiation, the cells were kept in medium containing 5% KnockOut serum replacement, 1% non-essential amino acids, and 2 mM L-glutamine (all Thermo Fisher) for another 10 weeks until they displayed pigmented cell patches. Immunohistochemistry (occluding) was performed on differentiated iPSC-derived RPE cells to confirm the RPE profile. For this purpose, cultures were fixed in 4% paraformaldehyde (ThermoFisher) and permealised for 10 min using 0.2% Triton-X in phosphate-buffered saline (PBS). The blocking was performed for 45′ in 10% normal goat serum in 0.2% Triton-X. Following overnight incubation of the first antibody (rabbit anti-occludin, ThermoFisher catalogue number 71–1500) in PBS-Triton-X, several washes in 0.025% Tween-20 were performed before administering the secondary antibody, goat anti-mouse Cy5 in PBS (Jackson). Following a 45′ incubation period and several washes in 0.025% Tween-20, cells were counter-stained using Hoechst (1:1000 in PBS). After an incubation of 20 min and a final washing step in PBS, the coverslip was mounted using fluorescent mounting media. 

Human primary fibroblasts (kindly provided by Dr. Dominik Waluk, DermFocus, Vetsuisse Bern, University of Bern) were cultured in high-glucose DMEM supplemented with P/S and 10% FBS and split 1:5 using TrypLE Express.

HEK 293T cells (ATCC CRL3216, a kind gift of PD Dr. P. Plattet, Division of Experimental Clinical Research, Vetsuisse Bern, Switzerland) were cultured and used to generate the CD63-GFP lentivirus. They were cultured in maintenance medium consisting of high-glucose DMEM supplemented with 10% FBS and 1% P/S.

### 4.2. Culture of T. gondii

Tachyzoites of *T. gondii* ME49 strain were cultured in ARPE-19 host cells at 37 °C and 5% CO_2_. They were maintained though serially passaged ARPE-19 cells. For this culture, the tachyzoites were harvested by the dissociation of the infected monolayer with a cell scraper, repeatedly passaged through a 25 G needle, and subjected to Sephadex G25 chromatography as described in [38,39]. The ARPE-19 cells were infected at a 1:1 ratio (parasites: host cells).

### 4.3. Exosome Isolation

#### 4.3.1. Urinary Small EVs

The first-void mid-stream urine sample of healthy volunteers was collected into plastic containers, and three protease inhibitors (cOmplete, Mini, Cocktail, Roche Applied Science, Mannheim, Germany) were added to 70 mL of urine. To remove contaminants, the urine samples were first centrifuged at 300× *g* (10 min) prior to a higher speed centrifugation step at 2000× *g* (10 min).

The urine samples were then clarified by vacuum filtration (TPP rapid Filtermax with 0.22 μm PVDF) before proceeding to a concentration step using a filter device (Centricon MWL 10,000 UFC701008, Merck Millipore, Darmstadt, Germany). For this concentration step, the precleared urine was centrifuged at 3220× *g* (Eppendorf centrifuge 5810R, Rotor A-4-62, Vaudaux-Eppendorf, Schönenbuch, Switzerland) according to the manufacturer’s instructions. The concentrate was eluted at a speed of 1000× *g* for 2 min. 

The collected small EVs were stored at −20 °C for further analysis.

The urinary small EVs were further purified using qEV exosome isolation size-exclusion chromatography (SEC) columns (Izon Science, Oxford, UK) according to the manufacturer’s instructions, and the eluted fractions were analysed for exosome size and concentration using nanoparticle tracking analysis (NTA) as described below.

#### 4.3.2. Cell Culture-Derived Small EVs

Conditioned medium (CM) from the CD63-GFP transduced human fibroblast culture was used to isolate secreted small EVs. For this purpose, human primary bone-marrow mononuclear cells were seeded in T300 flasks (TPP90301, Faust AG, Schaffhausen, Switzerland) at a density of 2 × 10^4^ cells/cm^2^. Media was changed every 3 days using prewarmed fibroblast medium. At 70% confluence, cells were washed twice in PBS to removed cellular debris before incubation for 3 days in before supernatant collection. The CM was first centrifuged at RT (300× *g*) to remove cellular contaminants prior to a centrifugation step at 2000× *g* followed by ultrafiltration as described above. The small EVs were stored at −20 °C, and the concentration was assessed using NTA (see below).

### 4.4. Exosome Characterization

#### 4.4.1. Antibody Array

The presence of known exosomal markers was assessed in urinary small EVs using Exo-Check antibody array (EXORAY200A-4-SBI, BioCat, Heidelberg, Germany) according to the manufacturer’s instructions. Frozen small EVs were thawed on ice, and 50 µg of each sample was lysed, labelled, and exposed to a membrane containing 12 spots with the 8 exosome marker antibodies (CD63, CD81, Alix, Flot1, ICAM1, EpCAM, ANXA5, and Tsg101) and 4 controls. Following the washing steps and signal detection (WesternBright Sirius chemiluminescent substrate, Biozym Scientific, Hessisch Oldendorf, Germany) according to the manufacturer’s instructions, the blots were imaged using a CCD camera on an Azure c600 Western blot imaging system (Axon Lab, Baden, Switzerland).

#### 4.4.2. Transmission Electron Microscopy (TEM)

Negative staining was performed on unlabelled urinary small EVs as well as on CD63-GFP tagged fibroblast-derived small EVs, which were fixed overnight in 4% paraformaldehyde (Thermo Fisher) in PBS. The fixed small EVs were pipetted onto carbon-coated 200-mesh copper grids, incubated for 5 min, washed for 5 min in water, and floated on 2% phosphotungstic acid at pH 7.0 (Merck, Darmstadt, Germany) for 45 s. The liquid was blotted with filter paper, and the grids were allowed to dry for 30 min. Then, the specimens were imaged using a Philips CM12 transmission electron microscope (FEI, Eindhoven, The Netherlands). Micrographs were captured with a MegaView III camera (Olympus, Münster, Germany) using iTEM software (version 5.2. Olympus Soft Imaging Solutions GmbH, Münster, Germany).

#### 4.4.3. Immunolabelling Using CD63 Antibody

The presence of the CD63 transmembrane protein on urinary small EVs was confirmed using immunogold-electron microscopy. For this procedure, extracellular vesicle suspensions were fixed with 2% paraformaldehyde and adsorbed to formvar/carbon-coated nickel grids for 5 min. The grids were then washed for 15 min in 0.05 M filtered glycine (Sigma-Aldrich, Buchs, Switzerland) in PBS.

Following incubation in washing buffer (0.1% bovine serum albumin (Sigma) in PBS, pH 7.4), the samples were blocked with 1% normal donkey serum (Jackson ImmunoResearch, West Grove, PA, USA) for 30 min. The grids were incubated overnight with a mouse anti-human CD63 antibody (1:100, DSHB hybridoma H5C6, deposited by August, J.T./Hildreth, J.E.K.) as described in [40]. Following several washes and incubation with 12 nm gold-conjugated donkey anti-mouse antibody (Jackson, diluted 1:20 in wash buffer) for 2.5 h, the grids were washed, post-fixed in 2.5% glutaraldehyde (Merck, Darmstadt, Germany) in PBS, and washed with PBS and distilled water. The excess fluid was blotted with filter paper, and the samples were dried for 30 min before TEM was carried out as described above.

#### 4.4.4. Nanoparticle Tracking Analysis (NTA)

The concentrations of BODIPY dye-labelled and unlabelled urinary small EVs in suspension, as well as the labelling efficiencies, were analysed using a NanoSight NS300 nanoparticle tracking device (Malvern Instruments Inc., Malvern, UK). Furthermore, size-distribution was also determined using the supernatant of CD63-GFP transduced fibroblasts. Briefly, exosome extracts were vortexed before diluting the small EVs in sterile PBS. Videos of the particles undergoing Brownian motion in a laser beam were recorded. Five videos (60 s each) were recorded per sample, and the mean was analysed to obtain the median exosome size. For each sample, the nanoparticle size distribution curve and the concentration of each particular size were recorded using NTA 2.3 software.

For BODIPY-labelled small EVs, the labelling efficiency was determined using the corresponding longpass filter (500 nm), which blocks the excitation wavelength (488 nm), thus allowing only the selective detection of the emission wavelength (called the fluorescent mode). A comparison of the vesicle counts obtained in both the fluorescent and light scatter modes (count of labelling efficiency) revealed the efficiency as percentages of fluorescent small EVs detectable with a NS300 nanoparticle tracking device.

### 4.5. Exosome-Labelling Procedures

#### 4.5.1. Labelling of Isolated Small EVs Using Lipophilic Dyes

The isolated urinary small EVs were labelled using two lipophilic dyes: PKH67 (Sigma) for the photooxidation experiment (due to the photostability of PKH) and BODIPY FL C_16_ (Thermo Fisher). The small EVs were incubated in 10 µM BODIPY in PBS for 30–40 min at 37 °C in 5% CO_2_. For the PKH67 labelling, the small EVs suspended in PBS were diluted 1:1 with 4 µM PKH67 that was prepared in dilution C buffer (provided in the PKH67 fluorescent cell linker kit, Sigma Aldrich, Buchs, Switzerland) and incubated at room temperature for 15 min. The reaction was stopped for both labelling reactions by adding an equal volume of 1% BSA (Sigma). Unincorporated dye was removed using Invitrogen™ spin columns (MW 3000, Thermo Fisher) according to the manufacturer’s instructions. Subsequently, the labelled small EVs were resuspended in ARPE-19 medium and tested for labelling efficiency or immediately transferred to the cell cultures for exosome uptake analysis. As a control, BODIPY alone was also column-purified and the eluate (after removal of unbound dye) was used to evaluate potentially residual unbound dye in the labelled experiment. 

#### 4.5.2. Genetical Labelling of Small EVs

Human fibroblasts expressing a GFP-tagged version of the transmembrane protein CD63 were produced. The CD63-GFP plasmid pCT-CD63-GFP (CYTO120-PA-1-SBI, System Biosciences, BioCat) was transformed into DH5α-competent *Escherichia coli* using standard techniques. Briefly, competent cells were thawed on ice for 20 min, and 20 µL of the bacterial suspension were mixed with 100 ng of the pCT-CD63-GFP plasmid and incubated on ice for 30 min before the bacteria were subjected to heat shock (42 °C, 20 s). The bacteria were then placed on ice for 5 min. Fifty microliters of competent cells were finally plated onto agar plates containing ampicillin (100 µg/mL) and incubated overnight. Colonies were selected for inoculation into LB broth (Lennox, Sigma Aldrich, Buchs, Switzerland) liquid microbial growth medium and further cultivated for 18 h with a 190 rpm shaking at 37 °C. Subsequently, the plasmid pCT-CD63-GFP was purified using the HiPure Plasmid Midiprep kit (Invitrogen, Thermo Fisher) according to the manufacturer’s instructions.

HEK293T cells were transfected with prepared plasmids to produce the CD63-GFP lentivirus. For this purpose, 9 million HEK293T cells were plated in a 10 cm dish in 293T medium without P/S overnight. The cells were then cotransfected with three vectors (3 µg of pMD2G envelope plasmid (Addgene plasmid # 12259 (Didier Trono); http://n2t.net/addgene:12259; RRID:Addgene_12259)), 8 µg of R8.91 packaging plasmid (generated by Didier Trono (Addgene plasmid # 22036; http://n2t.net/addgene:22036; RRID:Addgene_22036)), both kind gifts from M. Alves (Vetsuisse Faculty, University of Bern, Switzerland), and 10 µL of pCT-CD63-GFP transfer vector) using the standard calcium phosphate co-precipitation method. After cultivation at 37 °C and 5% CO_2_ for 24 h, the medium was replaced with fresh pre-warmed 293T medium. On days 2 and 3 post-transfection, the supernatant containing the virus particles was harvested, centrifuged at 500× *g* at 4 °C and passed through a 0.45 µm filter before freezing the lentivirus at −80 °C.

The virus was titrated using the qPCR lentivirus titration kit (Applied Biological Materials Inc., Richmond, ON, Canada) before transducing the human fibroblasts that were plated the day before (20,000 cells/cm^2^) with a multiplicity of infection (MOI) of 10 in medium containing 8 µg/mL polybrene for 16 h. The medium was changed, and puromycin selection was initiated at a concentration of 10 µg/mL. Three days later, the medium was changed with fresh puromycin-containing medium, and starting from day 7 post-transduction, the cells were split twice a week in puromycin-containing medium. The medium was changed every 3–4 days, and the transduced cells were split four times. After 3 passage, cells were plated and fed every 3 days with medium until 60% confluence before adding fresh medium (devoid of P/S) for 3 days. Thereafter, the secreted small EVs were isolated from the cell culture medium of the transduced fibroblasts by ultrafiltration as described above (Section 4.3.2).

### 4.6. Exosome Uptake and Imaging

ARPE-19 cells (Passage 27) were seeded on round glass coverslips (12 mm diameter, strength 1, Novoglas, Villmergen, Switzerland) in a 24-well plate (TPP, Faust Laborbedarf AG, Schaffhausen, Switzerland) at a concentration of 20,000 cells/cm^2^. At a confluence of 80%, they were incubated with BODIPY-labelled urine small EVs (0.5 × 10^9^ EVs/cm^2^) or CD63-GFP tagged EVs (8 × 10^8^) for 18 to 24 h. For the photooxidation experiment, PKH67-labelled small EVs were administered for 16 h to ARPE19 cells at a concentration of 2.8 × 10^9^ small EVs/cm^2^).

RPE cells that were differentiated from human induced pluripotent stem cells and that exhibited the typical in vivo-like cobblestone morphology were used to confirm the data obtained with the immortalized ARPE-19 cell line using the same concentration of EVs and treatment time as described for ARPE19 cells. 

After exosome uptake, all the cells and tissues were fixed for 1 h at RT in 4% paraformaldehyde before extensive washes in 1× PBS. 

Following permeabilization with 0.2% Triton X-100 in PBS, the cells on slides were counterstained using Hoechst dye (1 µg/mL, Sigma Aldrich, Buchs, Switzerland). The cover slips were mounted using Fluoromount (Sigma), and the samples were imaged using either an LSM 880 confocal microscope (Zeiss, Feldbach, Switzerland) or Axio Imager Z1 (Zeiss, Feldbach, Switzerland). Images were processed (z-projections or orthogonal views) using ImageJ software (Fiji, [41]).

Additional ARPE19 cells that were incubated overnight using BODIPY-labelled urinary small EVs were assessed for the colocalization of internalized small EVs and late endosomes/lysosomes using lysoTracker™ Deep Red (ThermoFisher) according to the manufacturers instruction. Lysotracker was added 16 h after incubating ARPE19 with labelled small EVs, following incubation for 1 h. Cells were fixed in 2% PFA (30 min), counterstained using Hoechst and actin, and imaged as described above.

### 4.7. Combination of Exosome Uptake and Toxoplasma Infection

ARPE-19 cells were seeded onto 8-well slides (ibidi µ-slide, Gräfelfing, Germany) at a density of 20,000 cells/cm^2^. After 8 h, the cells were infected with freshly purified *T. gondii* ME49 tachyzoites (2 × 10^4^ parasites per cm^2^). After 24 h, 10^8^ BODIPY-labelled urinary small EVs were added to each well and incubated for another 16 h. The specimens were fixed in freshly prepared 2% PFA (20 min, RT). Following washes in PBS, the cells were permeabilized in acetone:methanol (1:1) for 20 min at −20 °C. After washes in 0.025% Tween-20 in PBS, the infected cells were incubated overnight at 4 °C with a surface-antigen-1 mouse monoclonal antibody (anti-SAG-1, 1:100 in PBS) as described in [42]. Following several washes in PBS, the cells were incubated for 45 min with 1 mg/mL Cy3-conjugated goat anti-mouse IgG antibody (1:400 dilution, Jackson ImmunoResearch 115-165-062, Cambridgeshire, UK). Nuclear counterstaining was performed using Hoechst (1 µg/mL. Sigma). Thereafter, the slides were mounted in Fluoromount (Sigma), placed onto glass slides and imaged using a confocal microscope (Zeiss LSM 880, Feldbach, Switzerland).

### 4.8. Photooxidation

ARPE-19 cells were grown on sterilized round glass coverslips (12 mm diameter, strength 1, Novoglas, Villmergen, Switzerland) and exposed to PKH67-labelled small EVs (2 × 10^9^ particles/cm^2^) for 18 h at 37 °C and 5% CO_2_. Following washing (3 × 5 min) in pre-warmed (37 °C) 1 × PBS, the cells were fixed in a solution containing 0.5% glutaraldehyde (Merck, Darmstadt, Germany) and 4% PFA (Thermo Fisher) in PBS for 30 min at 37 °C. After the cells were washed in PBS, nuclei were counterstained using Draq5 (30 µM final concentration, Thermo Fisher) for 15 min at RT. Following several washes in PBS, the cells were maintained in 0.05 M Tris buffer (pH 7.4) at 4 °C until photoconversion. The cover slips, cells facing down, were mounted on a paper clip that was glued to a glass slide.

The glass slide was held on a Peltier element-based cooling device set at a temperature of 10 °C, as described in [43], to minimize the spontaneous oxidation of 3,3′-diaminobenzidine (DAB) and diffusion of the reaction product and to increase the saturation point of the dissolved oxygen.

Using an Axio Imager microscope (Axio Imager Z1) connected to a powerful light source (X-Cite XYLIS LED fluorescence light source, Excelitas, Waltham, MA, USA) equipped with a 488-filter set. The regions of interest were determined before changing the medium to 3,3′-diaminobenzidine (DAB D-8001, Sigma Aldrich, Buchs, Switzerland) solution (2 mg/mL), freshly prepared in 0.05 M Tris buffer at pH 7.4 and filtered using a CHROMAFILXtra RC-45/25 filter (Macherey Nagel, Oensingen, Switzerland). Thereafter, the fluorochrome of the second antibody was excited for the photoconversion of DAB. Illumination was maintained for 15 min using a 20× objective (Plan-Apochromat 20×/0.8 NA, Zeiss, Germany) and the respective FITC filter set. The reaction was stopped when a brown precipitate was observable when exchanging the DAB solution with fresh Tris buffer pH 7.4.

After photooxidation, the bleached area was imaged to ensure photobleaching and complete DAB photoconversion. The cells were then washed in 0.1 M cacodylate buffer and post-fixed overnight with 2.5% glutaraldehyde in 0.1 M cacodylate buffer at 4 °C. Following further washing steps in ddH_2_O, the cells were osmicated with 1% OsO_4_ (Chemie Brunschwig, Basel, Switzerland) in 0.1 M sodium cacodylate, pH 7.4, and were washed again in cacodylate buffer. Thereafter, the cells were dehydrated in an ascending ethanol series (70%, 80%, 90%, and twice at 100% for 20 min at each concentration) and embedded in Epon 812 epoxy resin (Sigma-Aldrich Chemie GmbH, Buchs, Switzerland). Following resin polymerization at 60 °C for 48 h, ultrathin sections were cut with a diamond knife (Diatome, Biel, Switzerland) on a Reichert-Jung Ultracut ultramicrotome (Leica Microsystems, Heerbrugg, Switzerland) before collection on collodion-coated copper grids (Electron Microscopy Sciences, Hatfield, PA, USA). The sections were stained with UranyLess (Electron Microscopy Sciences) and 3% lead citrate (Laurylab, Saint Fons, France) before examination using a Philips CM12 transmission electron microscope (FEI, Eindhoven, The Netherlands) at an acceleration voltage of 80 kV. Micrographs were captured as described above. As controls (no photobleaching, no background DAB signal), the regions incubated in DAB without being illuminated were cut and imaged as well.

## Supplementary Materials

Supplementary materials can be found at https://www.mdpi.com/1422-0067/21/11/3799/s1.
