# Supplementary material for: Isolation of Human Small Extracellular Vesicles and Tracking of Their Uptake by Retinal Pigment Epithelial Cells In Vitro
Source: Int J Mol Sci. 2020 May 27;21(11):3799. doi: 10.3390/ijms21113799 (PMC7313035; doi:10.3390/ijms21113799)
Supplement: Supplementary file 1 [file ijms-21-03799-s001.zip › Supplementary Figures Final.pdf]

International Journal of *Molecular Sciences*

Supplementary Figures

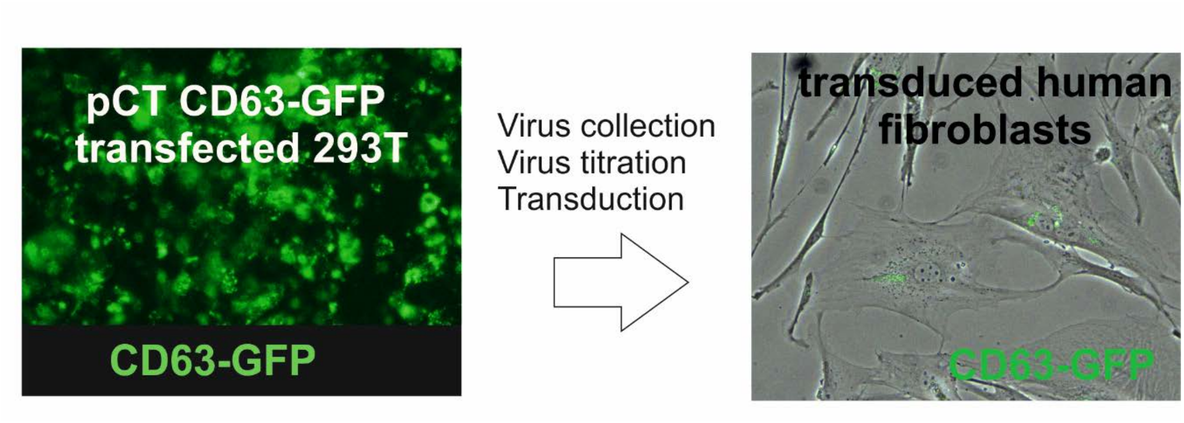

Figure S1. Generation of human primary cells releasing fluorescent small EVs. Transient CD63-GFP lentivirus production was performed in HEK293T cells (left side). Viral particles in the supernatant were collected, centrifuged and stored at -80°C prior to titre determination. Then, the primary human fibroblasts were transduced (MOI=10). The majority of transduced human primary fibroblasts displayed GFP-positive dots, indicating the exosomal CD63 tetraspanins tagged by GFP. The supernatant of these cells was ultrafiltrated and assessed using NTA.

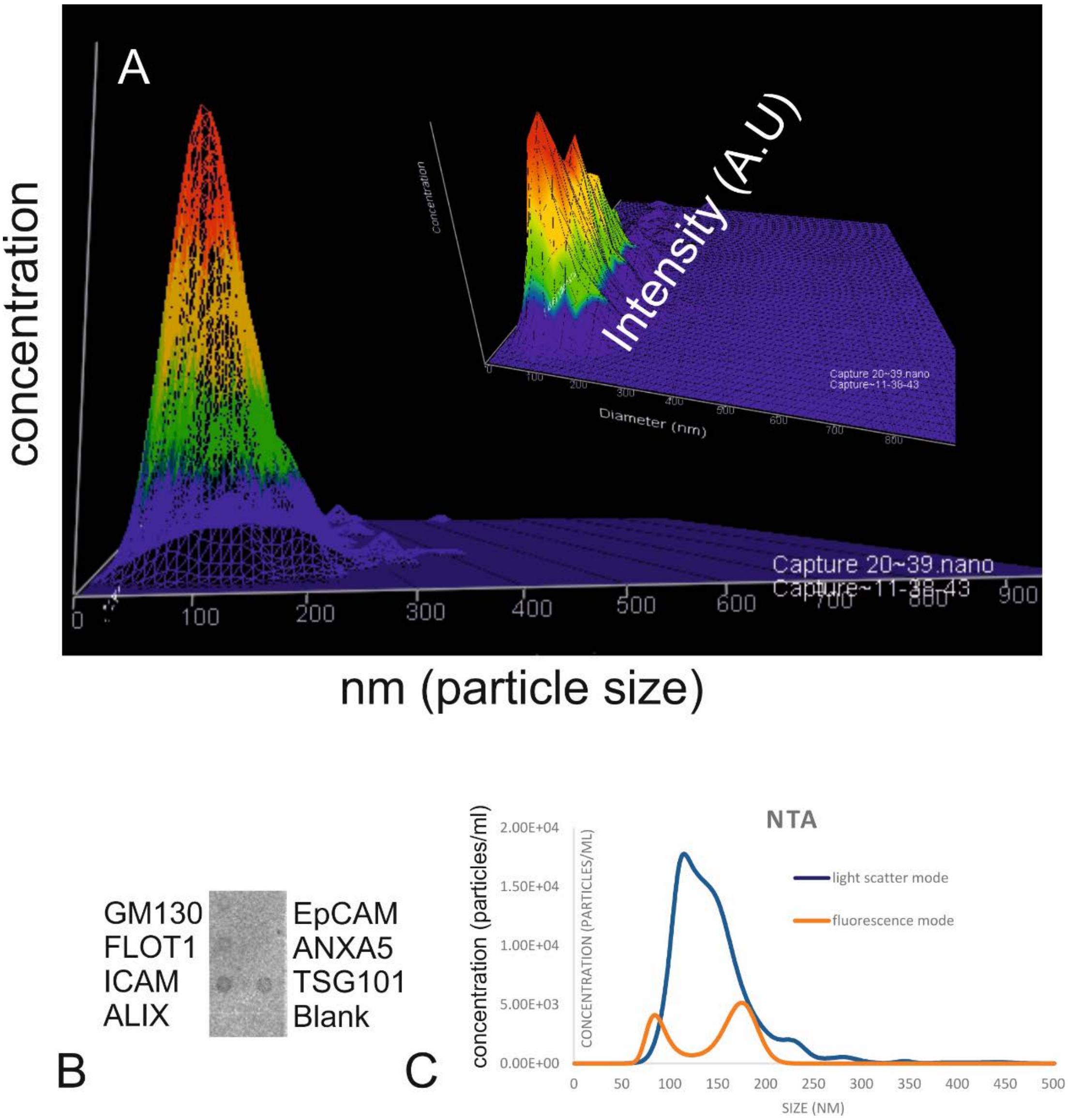

Figure S2. Characterization of CD63-GFP exosomes derived from virus-transduced human fibroblasts. (A) Nanoparticle tracking analysis of the supernatant of CD63-GFP transduced human fibroblasts. Refractive index 3D plots of the size versus intensity versus vesicle concentration showing that the small EVs isolated from suspension were monodisperse fractions with a peak mean size of 146 nm (10% of vesicles are smaller than 102 nm and 10% are bigger than 190 nm), although there were significant differences in size, intensity and concentration of the vesicles (smaller insert). (B) Dot-blot (array) indicating that obtained vesicles expressed exosomal markers (TSG101, ICAM, FLOT1). (C) NTA of GFP-tagged exosomes in the light scatter (blue line) as well as the fluorescent (red line) mode showing a dim but detectable fluorescence as opposed to unlabelled exosomes which do not show any background fluorescence (not shown).

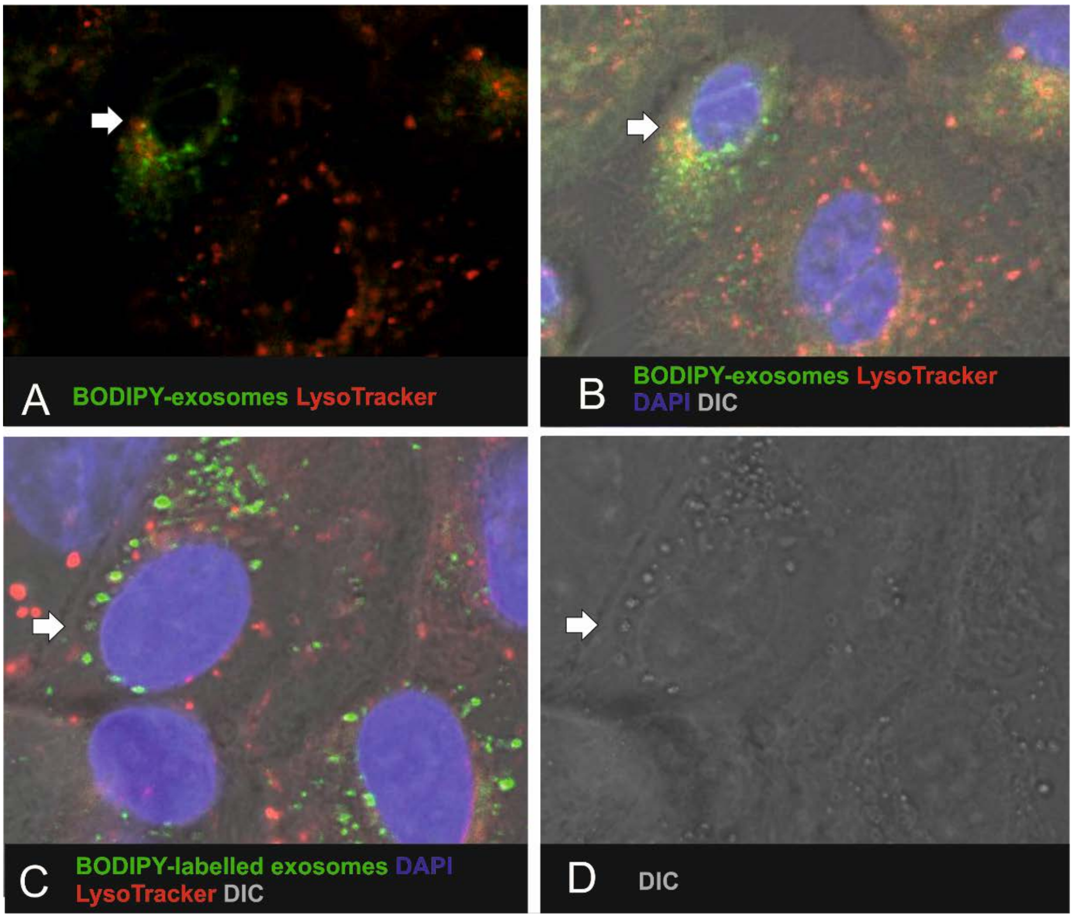

Figure S3. Co-localization of internalized BODIPY-labelled urinary EVs (green) and lysosomes (red). (A) 16 hours after the incubation of ARPE19 cells with BODIPY-labelled urinary small EVs, co-localization of fluorescent small EVs and lysosomes (lysotracker) was low (arrow). (B) DAPI counterstain showing nuclei and DIC displaying cellular outlines. (C) Higher magnification of Lysotracker (red) and small EVs (green) where no colocalization was visible. (D) Fluorescent-labelled EVs that were internalized are enclosed in larger contrast rich endocytotic intracellular vesicles (arrows).

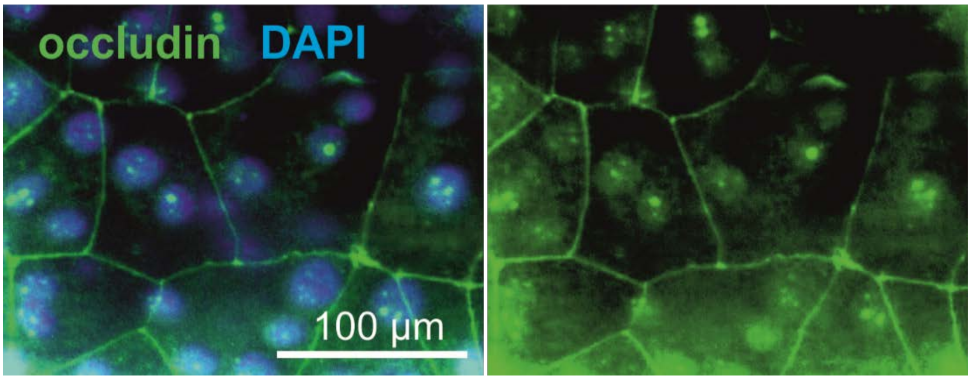

Figure S4. iPSC-derived RPE cells showed a typical cobblestone-like pattern with cells expressing occulin (green) as a tight-juction marker.

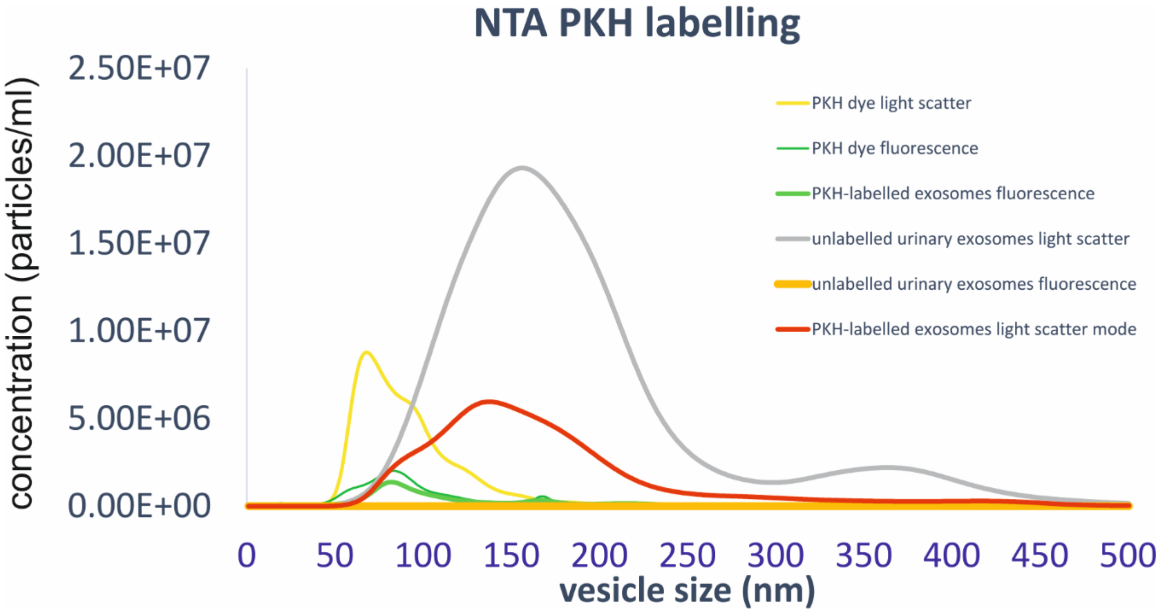

Figure S5. NTA of PKH dye, unlabelled urinary exosomes as well as PKH-labelled urinary exosomes in both light-scatter as well as fluorescent mode. Unlabelled urinary exosomes displayed a mean size of 189nm (grey line), with no fluorescence background detectable in the fluorescence mode (orange line). PKH labelling did not result in a size-shift (171nm mean size of vesicles, see light scatter mode: red line). When comparing the concentration of vesicles observed in the labelled urinary sample under the fluorescent mode (light green, mean size: 148nm) compared to the red line (total number of vesicles revealed that vesicles were only partially labelled (or fluorescence was too weak to be detected)). As a control, dye only controls (without column purification) revealed that dye particles show a size distribution of 90nm (yellow line), along with a weak but detectable fluorescence (green line).
