# Supplementary figures and images for: Isolation of Human Small Extracellular Vesicles and Tracking of Their Uptake by Retinal Pigment Epithelial Cells In Vitro
Source: Int J Mol Sci. 2020 May 27;21(11):3799. doi: 10.3390/ijms21113799 (PMC7313035; doi:10.3390/ijms21113799)

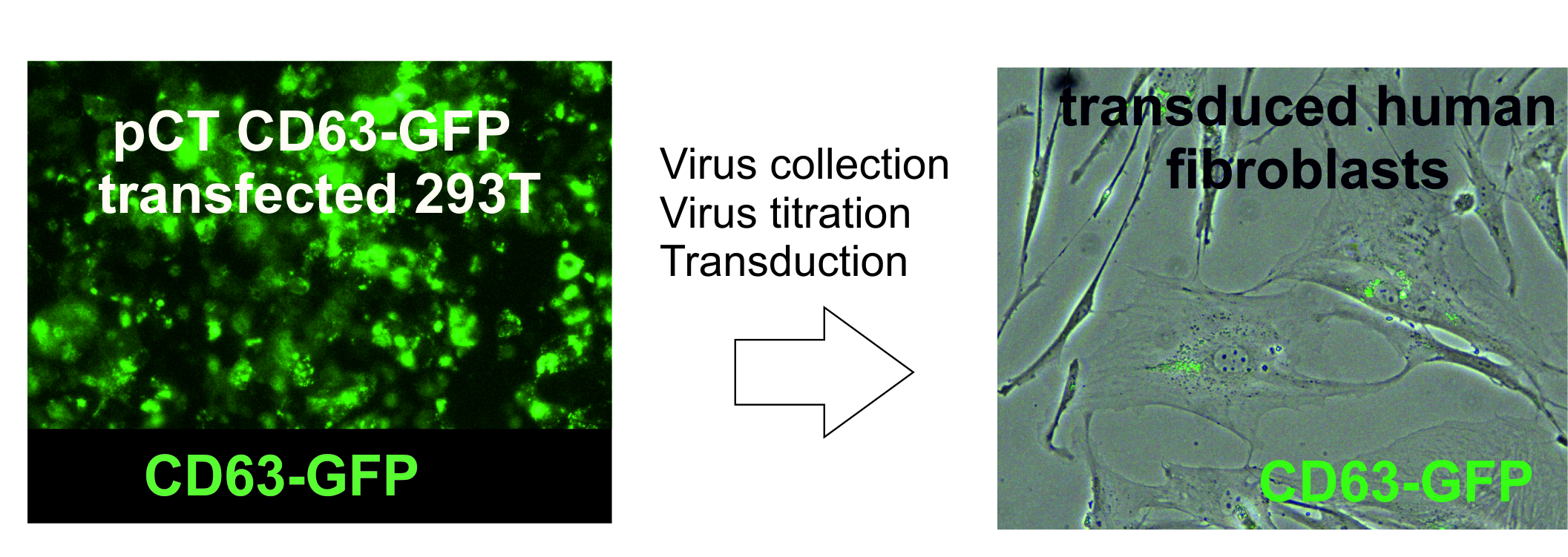

Supplement: Supplementary file 1 [file ijms-21-03799-s001.zip › Supplementary Fig1.tif]

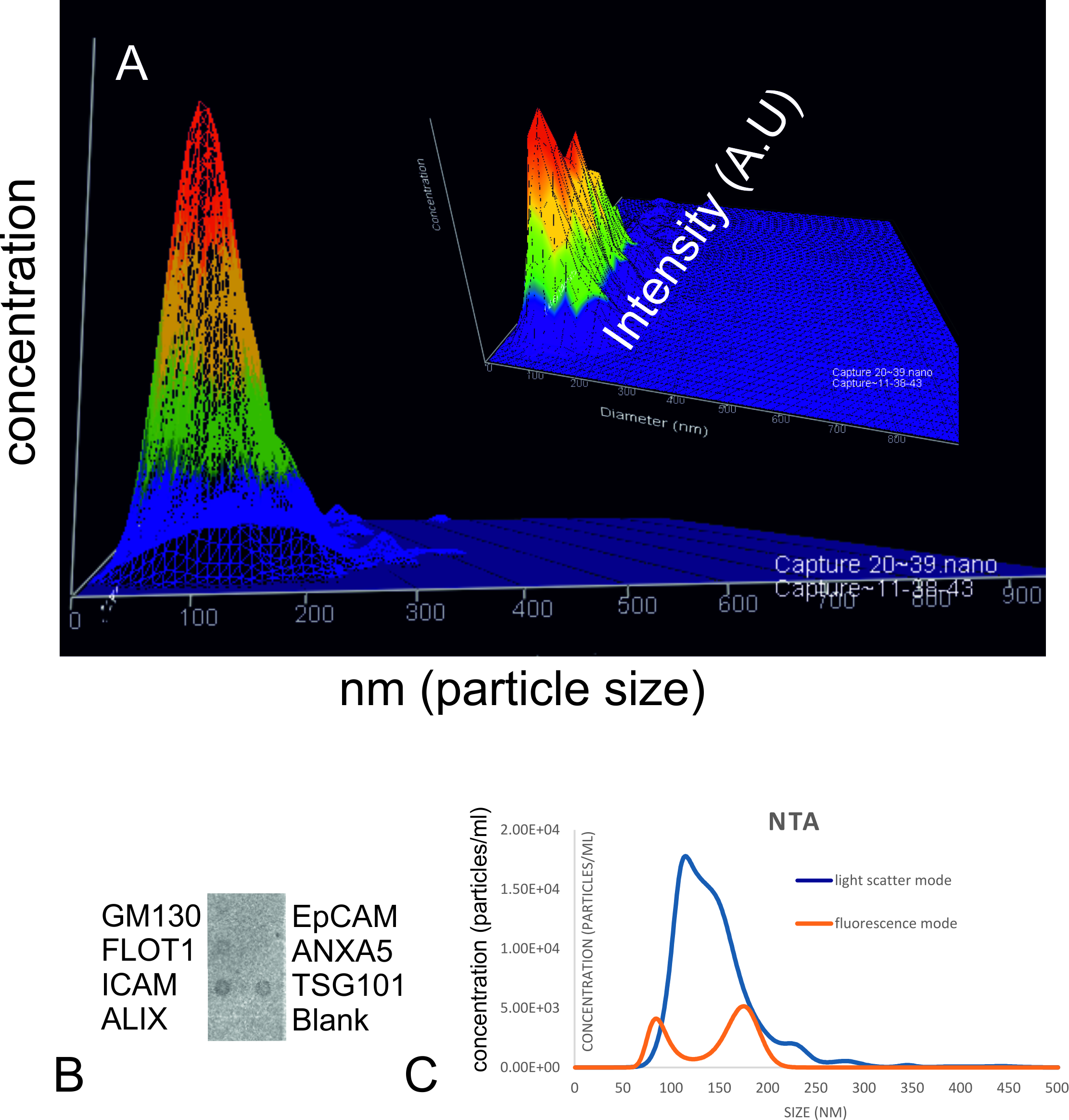

Supplement: Supplementary file 1 [file ijms-21-03799-s001.zip › supplementary Fig2.tif]

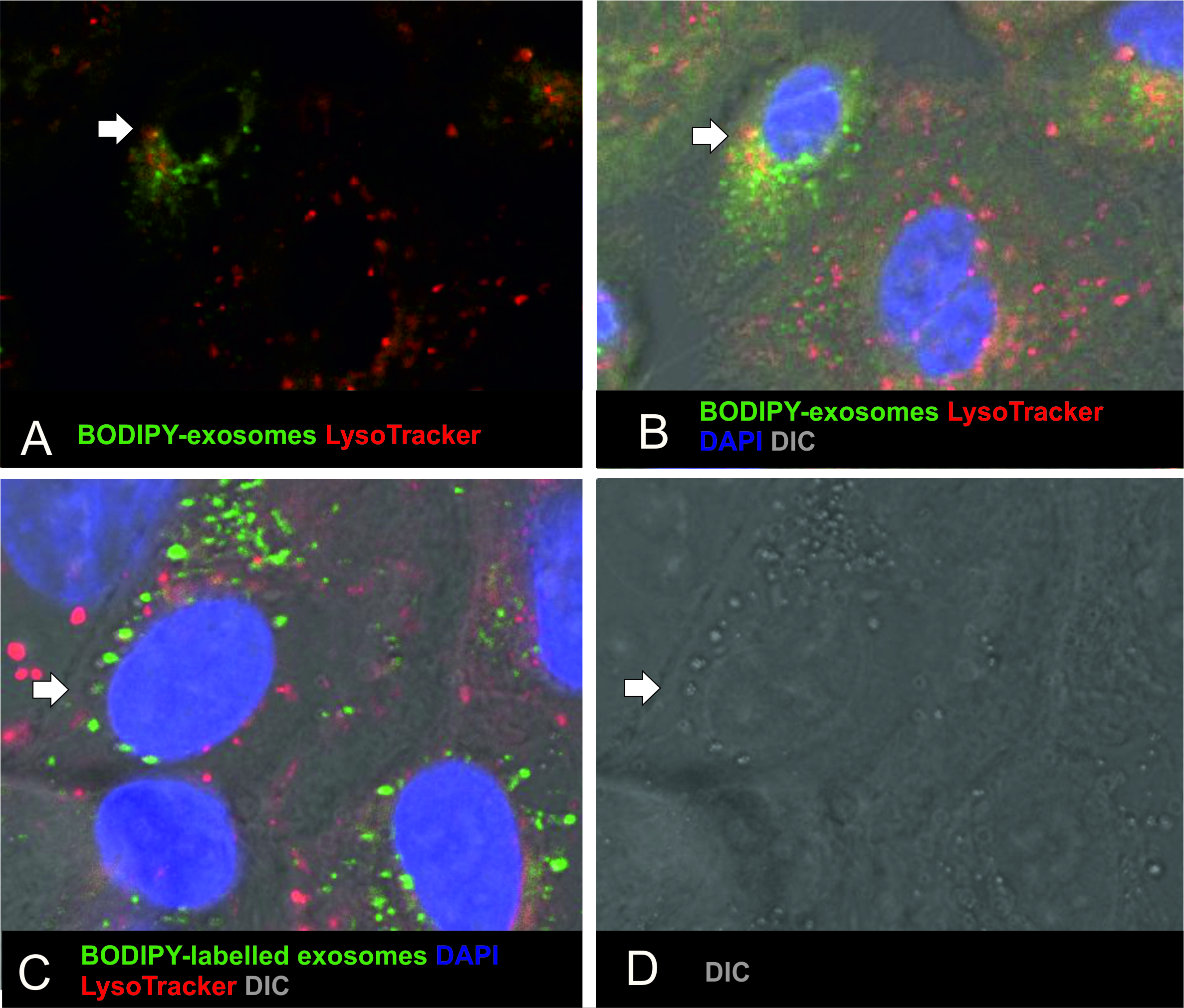

Supplement: Supplementary file 1 [file ijms-21-03799-s001.zip › Supplementary Fig3.tif]

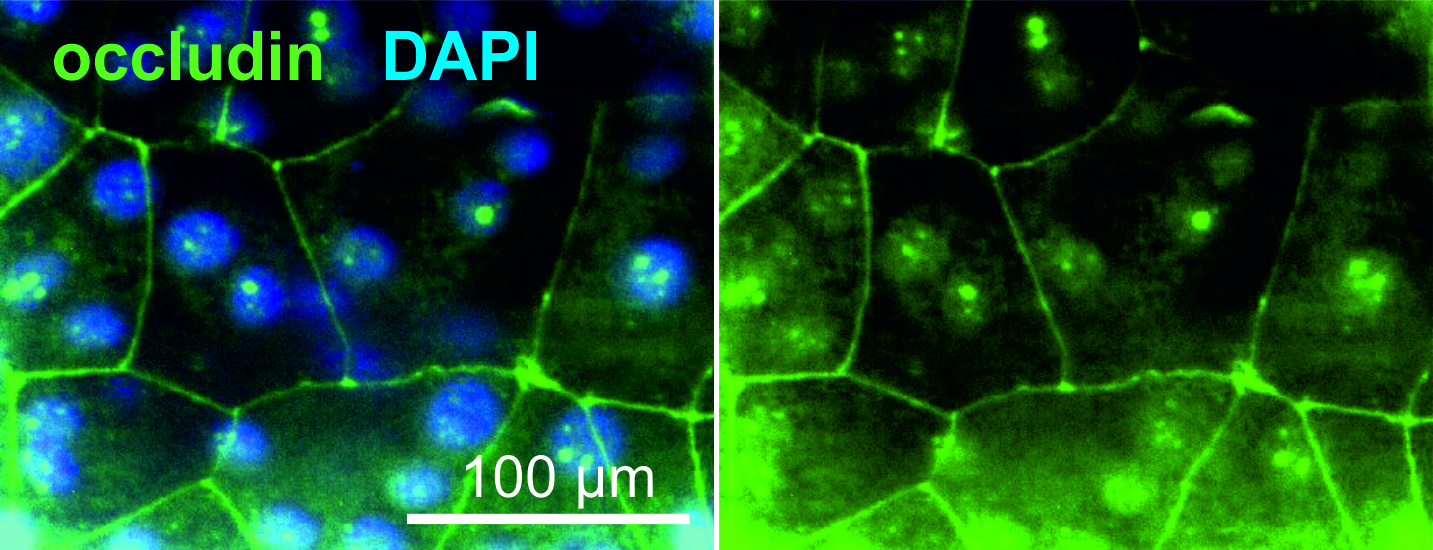

Supplement: Supplementary file 1 [file ijms-21-03799-s001.zip › supplementary Fig4.tif]

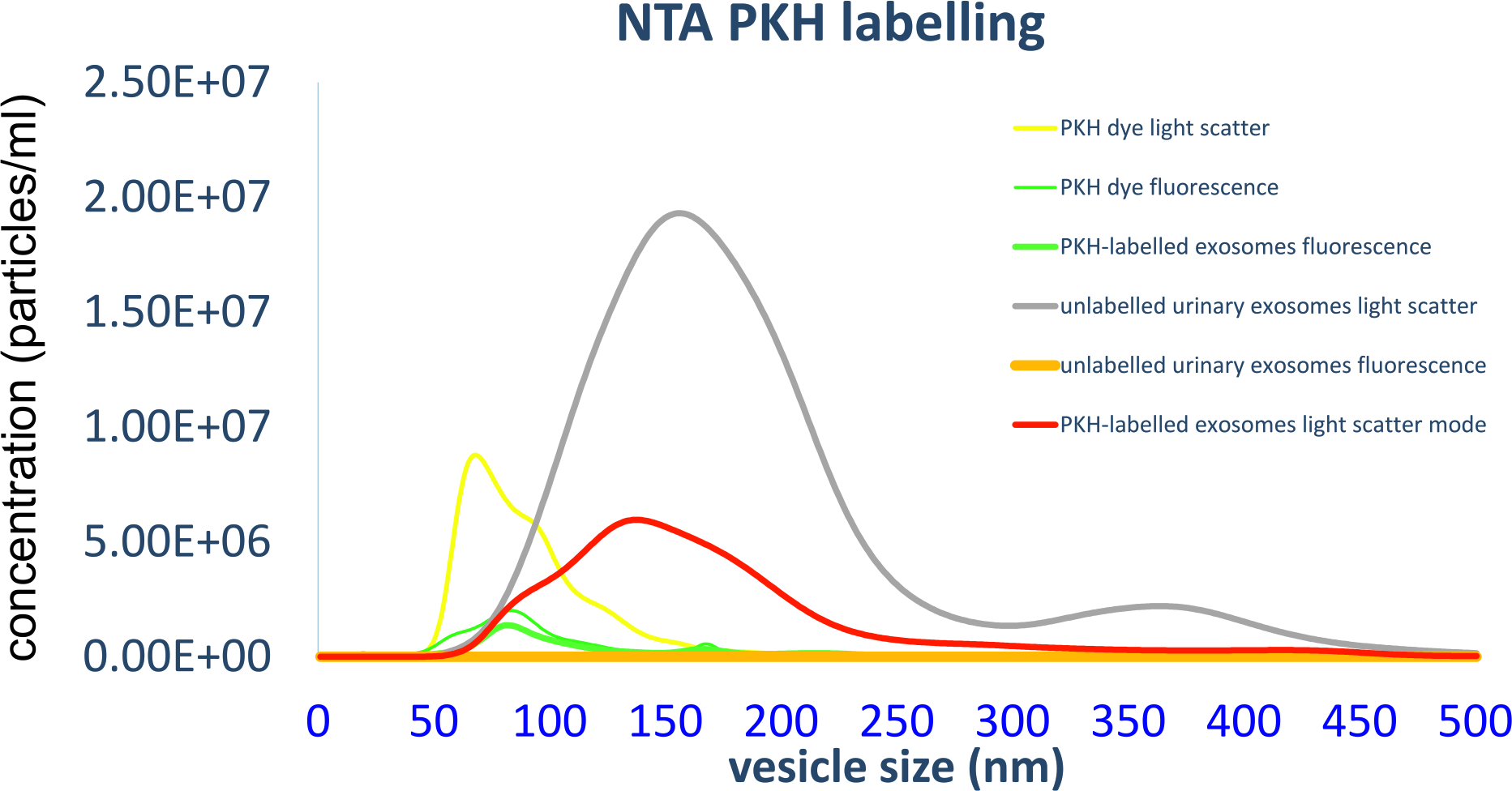

Supplement: Supplementary file 1 [file ijms-21-03799-s001.zip › Supplementary Fig5.tif]
